# Interleukin‐34 mediated by hepatitis B virus X protein via CCAAT/enhancer‐binding protein α contributes to the proliferation and migration of hepatoma cells

**DOI:** 10.1111/cpr.12703

**Published:** 2019-10-16

**Authors:** Fanyun Kong, Kai Zhou, Ting Zhu, Qi Lian, Yukai Tao, Nan Li, Tao Tu, Yanwei Bi, Xiaoying Yang, Xiucheng Pan, Shibao Li, Hongjuan You, Kuiyang Zheng, Renxian Tang

**Affiliations:** ^1^ Department of Pathogenic Biology and Immunology Jiangsu Key Laboratory of Immunity and Metabolism Xuzhou Medical University Xuzhou China; ^2^ National Demonstration Center for Experimental Basic Medical Sciences Education Xuzhou Medical University Xuzhou China; ^3^ Clinical Laboratory Enze Hospital Taizhou Enze Medical Center Luqiao China; ^4^ Department of Infectious Diseases The Affiliated Hospital of Xuzhou Medical University Xuzhou China; ^5^ Department of Laboratory Medicine The Affiliated Hospital of Xuzhou Medical University Xuzhou China

## Abstract

**Objectives:**

Interleukin‐34 (IL‐34) is associated with hepatitis B virus (HBV) infection and hepatocellular carcinoma (HCC). However, the role and associated mechanisms of IL‐34 in HBV‐related HCC remain unclear. In this study, the expression, biological function and associated mechanisms of IL‐34 in HBV‐related HCC cells were investigated.

**Methods:**

IL‐34 expression induced by HBV and HBV X (HBX) gene was measured in hepatoma cells. The role of CCAAT/enhancer‐binding protein α (CEBP/α) in HBX‐induced IL‐34 expression was examined. The signal pathways involved in the expression of CEBP/α and IL‐34 induced by HBX were assessed. The role of IL‐34 in the proliferation and migration of HCC cells, and related mechanisms were explored.

**Results:**

Dependent on HBX, HBV increased IL‐34 expression in hepatoma cells, and HBX upregulated and interacted with CEBP/α to enhance the activity of IL‐34 promoters. CEBP/α mediated by HBX was associated with the activation of PI3‐K and NF‐κB pathways to promote IL‐34 expression. Via CSF1‐R and CD138, IL‐34 promoted the proliferation and migration of hepatoma cells, and contributed to the activation of ERK and STAT3 pathways and the upregulation of Bcl‐xl and c‐Myc mediated by HBX.

**Conclusion:**

We demonstrate that IL‐34 contributes to HBX‐mediated functional abnormality of HCC cells and provides a novel insight into the molecular mechanism of carcinogenesis mediated by HBX.

## 1 INTRODUCTION

Hepatitis B virus (HBV) is one of the most vital aetiological factors for the occurrence and progression of hepatocellular carcinoma (HCC).^1^, ^2^ However, the molecular mechanisms of hepatocarcinogenesis mediated by the virus are not well clarified. HBV genome contains four open reading frames (ORF): S, P, C and X. S ORF has HBS, preS1 and preS2 genes that encode three viral envelope proteins. P ORF encodes viral polymerase (HBP). The C ORF contains C and precore genes that responsible for the expression of viral core protein (HBC) and HBe protein. X is the smallest ORF that encodes HBV X protein (HBX). Among viral proteins encoded by HBV genome, HBX is considered as a cancer cofactor and modulates tumorigenesis via the regulation of expression and activity of multiple host factors.^1^, ^3^, ^4^, ^5^, ^6^ Especially, current studies indicate that HBX is capable of regulating various cytokines, including IL‐6,^7^ IL‐12^8^ and TGF‐β,^9^ to mediate the proliferation, apoptosis and migration of HBV‐related HCC. Further exploring the role and related mechanisms associated with the cytokines mediated by HBX will help us identify new therapeutic targets to improve the outcomes of HCC patients with HBV infection.

Interleukin‐34 (IL‐34) is a newly identified cytokine from a comprehensive human protein library.^10^ Binding to three receptors, including colony‐stimulating factor 1 receptor (CSF1‐R), CD138 and PTP‐ζ,^11^ IL‐34 could regulate the differentiation and function of various target cells. Until now, collective evidence has demonstrated that IL‐34 is involved in the development of viral infection, autoimmune diseases and cancers.^12^, ^13^ Importantly, recent studies show that IL‐34 is involved in the HBV infection and associated with liver fibrosis.^14^, ^15^ Besides, the report from Zhou S et al shows that increased IL‐34 is related to the poor survival and tumour recurrence in HCC patients, and modulates the invasion and metastasis of HCC cells via macrophages.^16^ However, whether IL‐34 contributes to the development of HBV‐infected HCC is still unclear.

In this study, we investigated the expression, biological function and associated mechanisms of IL‐34 in HBV‐related hepatoma cells. We found that, in HBV associated HCC cells, via a transcription factor CCAAT/enhancer‐binding protein α (CEBP/α), HBX contributed to the increase of IL‐34. In addition, IL‐34 mediated by HBX contributes to the proliferation and migration of HCC. These results could improve our understanding on the underlying mechanism of hepatocarcinogenesis mediated by HBX during HBV infection.

## 2 MATERIALS AND METHODS

The source and culture of HepG2, Huh7 and HepG2.2.15 cells were described previously.^17^, ^18^ See the Supplementary Information for details regarding reagents, plasmids and clinical samples, and other materials and methods used in the study.

## 3 RESULTS

### 3.1 HBX is responsible for IL‐34 expression in HBV‐related HCC cells

**Figure 1:**
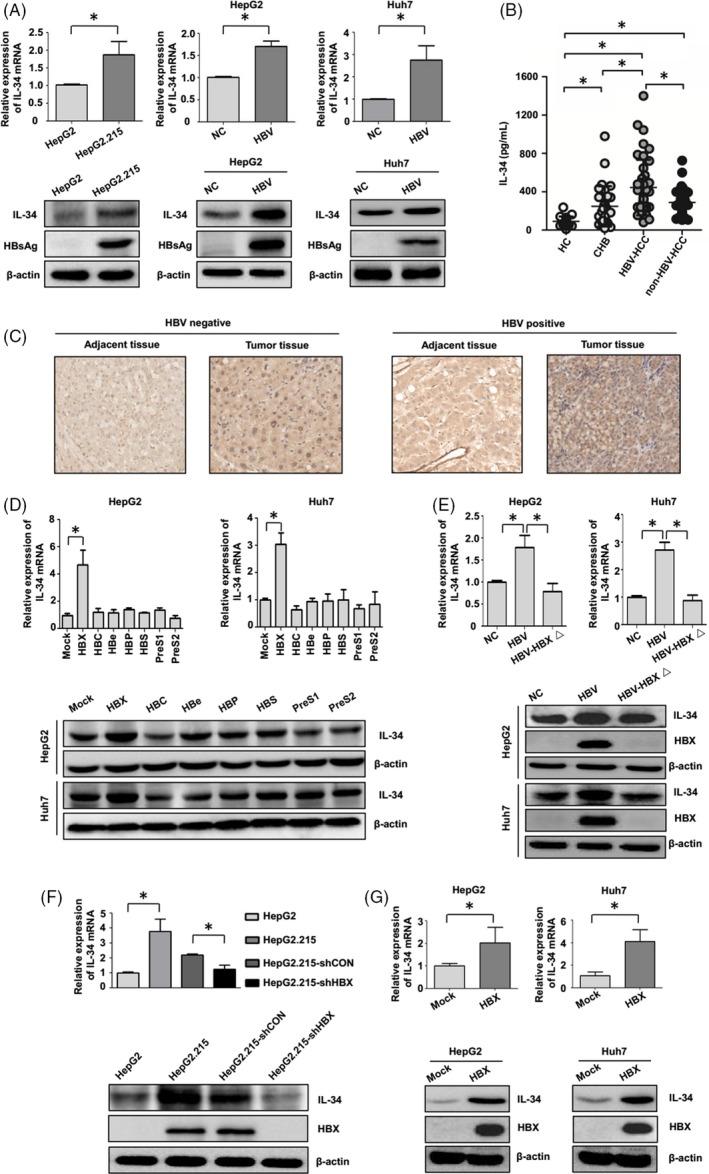
The role of HBX on expression of IL‐34 in HBV‐related HCC cells. A, The role of HBV on expression of IL‐34 in HCC cells in both mRNA and protein levels. B, The serum levels of IL‐34 in health controls (HC), CHB patients, HCC patients with HBV infection (HBV‐HCC) and HBV‐negative HCC patients (non‐HBV‐HCC) were detected by ELISA. C, The expressions of IL‐34 protein in HBV‐negative adjacent tissues, HBV‐negative HCC, HBV‐related adjacent tissues and HBV‐related HCC were detected by IHC analysis (200 × magnification). D, The role of encoding genes in HBV genome on IL‐34 mRNA and protein expression in HCC cells. E, The effect of HBV on IL‐34 expression in HCC cells with or without HBX gene. F, The role of HBV on expression of IL‐34 with the inhibition of HBX using shRNA plasmids. G, The expression of IL‐34 in HBX stably transfected HCC cells. NC: the HCC cells transfected with control plasmid; HBV: the HCC cells transfected with HBV plasmid. HBV‐HBXΔ: The cells transfected with HBV plasmid containing HBX deletion mutant. HepG2.215‐shCON: HepG2.215 transfected with shRNA control plasmid. HepG2.215‐shHBX: HepG2.215 transfected with shRNA plasmid against HBX. Mock: the cells transfected with negative control plasmid. HBX: the cells transfected with HBX plasmid. *P < .05

To investigate whether HBV could promote IL‐34 expression in HCC cells, the expression level of IL‐34 was measured in HepG2 and HepG2.215 cells (HepG2 cells with HBV genome). Compared with HepG2 cells, the expression of IL‐34 was increased in HepG2.215 cells (Figure 1A). Next, HBV and control plasmids were transfected into HepG2 and Huh7 cells, and we found that HBV could increase IL‐34 expression in both two types of hepatoma cells (Figure 1A). We evaluated serum IL‐34 levels in chronic hepatitis B (CHB) patients, HBV‐related HCC patients and HBV‐negative HCC patients. The results showed that the levels of serum IL‐34 were significantly higher in HBV‐related HCC patients than those in CHB and HBV‐negative HCC patients (Figure 1B). IL‐34 protein expression in HBV‐negative HCC, HBV‐negative adjacent tissues, HBV‐positive tumour tissues and HBV‐positive adjacent tissues were examined. Compared with HBV‐negative adjacent tissues, HBV‐positive adjacent tissues and HBV‐negative HCC, the expression of IL‐34 was elevated in HBV‐related tumour tissues (Figure 1C).

We next investigated the role of different HBV genes on IL‐34 expression, and only HBX was found to significantly increase IL‐34 expression in HCC cells (Figure 1D). In addition, we transfected hepatoma cells with HBV plasmids or HBV plasmids with HBX mutation to further detect the effect of HBX on IL‐34 expression in HCC cells with HBV infection. When HBX was deleted in the HBV genome, the expression of IL‐34 mediated by the virus was significantly inhibited in HCC cells (Figure 1E). We transfected HepG2.215 cells with HBX shRNA vectors and found that the knockdown of HBX could attenuate IL‐34 expression in HepG2.215 cells (Figure 1F). We also explore the expression of IL‐34 in hepatoma cells stably transfected with HBX or control plasmids. Compared with control cells, IL‐34 expression was elevated in HBX stable expressing cells (Figure 1G). Taken together, these results suggest that, dependent on HBX, HBV could facilitate IL‐34 expression in HCC cells.

### 3.2 HBX activates IL‐34 promoter through CEBP/α in hepatoma cells

**Figure 2:**
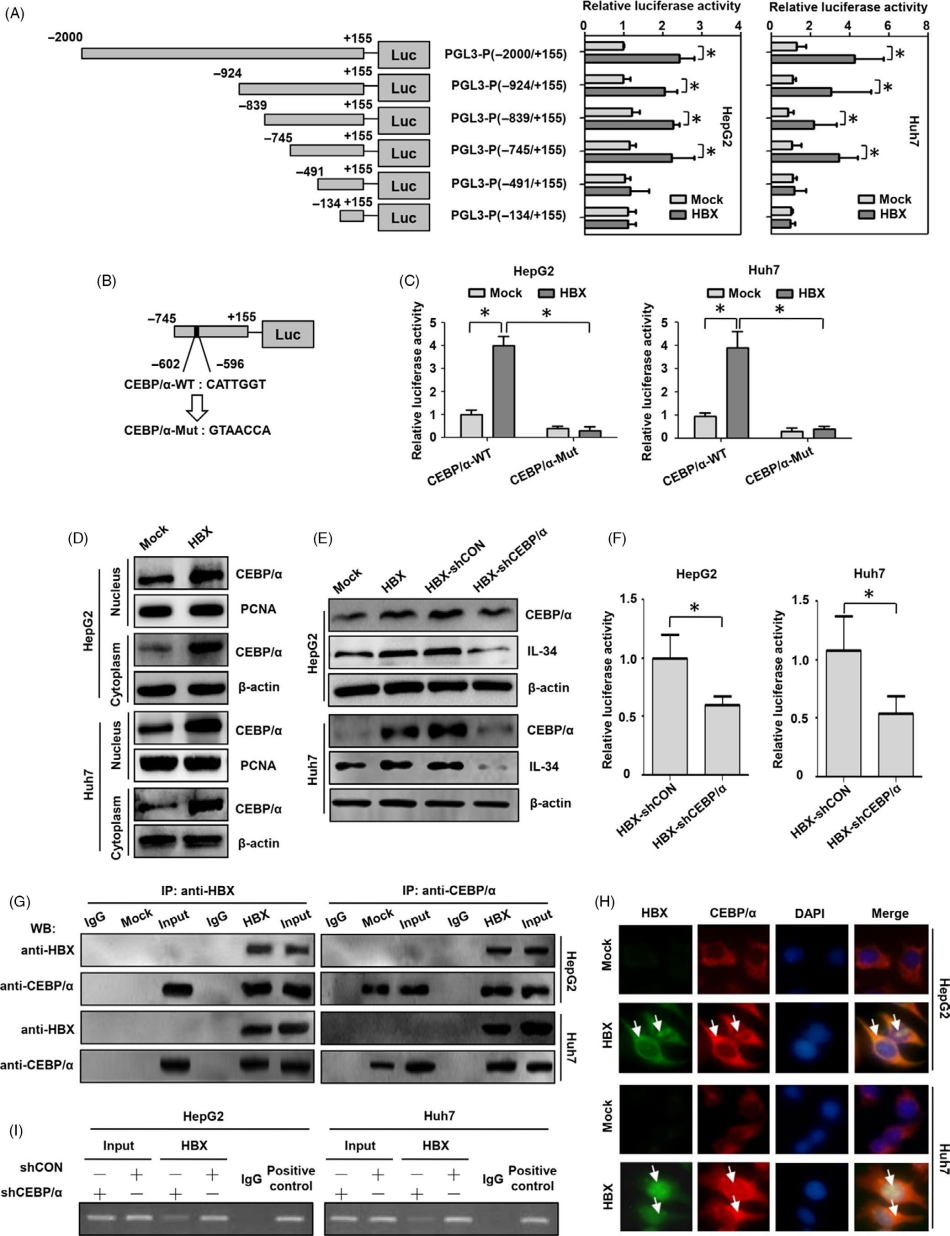
The effect of CEBP/α in activating the promoter of IL‐34 mediated by HBX in HCC cells. A, The identification of the regulatory region of IL‐34 promoter mediated by HBX via luciferase reporter gene assays. B, The mutation information of CEBP/α binding site in core region of the IL‐34 promoter in PGL3‐P (−745/+155) plasmid. C, The influence of the mutation in the CEBP/α binding site on activation of the core region of the IL‐34 promoter in PGL3‐P (−745/+155) plasmid. D, The expression of CEBP/α protein in the cytoplasm and nucleus of HBX‐positive HCC cells. E, The inhibition of CEBP/α using shRNA plasmids on the expression of IL‐34 protein. F, The inhibition of CEBP/α using shRNA plasmids on the activity of the core region of the IL‐34 promoter in PGL3‐P (−745/+155) plasmid. G, The interaction of HBX with CEBP/α using Co‐IP assay. H, The collocation of HBX (green) and CEBP/α (red) in HCC cells. The white arrows show the colocalization of HBX and CEBP/α in HCC cells. I, The role of CEBP/α in interaction between HBX and IL‐34 promoter as determined by ChIP assay. Mock: cells transfected with the negative control plasmids. HBX: cells transfected with HBX plasmids. CEBP/α‐WT: the CEBP/α site in the wild type, the CEBP/α‐MUT: the CEBP/α site with a mutation. HBX‐shCON: The HBX‐positive cells transfected with control plasmids. HBX‐shCEBP/α: The HBX‐positive cells transfected with shRNA plasmids against CEBP/α. *P < .05

To elucidate the mechanisms related to HBX‐mediated expression of IL‐34, six IL‐34 promoters fragments, including −2000/+155 (PGL3‐P[−2000/+155]), −924/+155 (PGL3‐P[−924/+155]), −839/+155 (PGL3‐P[−839/+155]), −745/+155 (PGL3‐P[−745/+155]), −491/+155 (PGL3‐P[−491/+155]) and −134/+155 (PGL3‐P[−134/+155]) were cloned into PGL3 plasmids, and these plasmids were cotransfected with HBX or control plasmids into HCC cells to examine the role of HBX on different fragments of the IL‐34 promoter. The results showed that (Figure 2A), HBX could activate the luciferase activities of PGL3‐P(−2000/+155), PGL3‐P(−924/+155), PGL3‐P(−839/+155) and PGL3‐P(−745/+155), but not PGL3‐P(−491/+155) and PGL3‐P(−134/+155). These results suggest that the regulatory site mediated by HBX is located in the region (−745/−491) of IL‐34 promoter.

The transcription factor binding sites in IL‐34 promoter regions (−745/−491) were predicted using tfsearch and alibaba2.0 software.^17^ The binding site of CEBP/α was found to locate in the promoter region (−745/−491) of IL‐34. CEBP/α is associated with the dysregulation of different genes mediated by HBX.^19^, ^20^ We assumed that HBX might activate the promoter of IL‐34 via CEBP/α. As expected, we found that the promoter activity of PGL3‐P(−745/+155) was inhibited when CEBP/α binding site sequences (−602/−596) were mutated in HCC cells (Figure 2B,C).

We examined the expression of CEBP/α in control cells and HBX‐positive HCC cells. Compared with control cells, CEBP/α expression in the nucleus and cytoplasm was increased in HBX‐positive HCC cells (Figure 2D). To further explore whether HBX activates the transcription of IL‐34 through CEBP/α, specific shRNA plasmids targeting CEBP/α were transfected into HBX‐positive cells. The results showed that the inhibition of CEBP/α could significantly suppress IL‐34 expression and IL‐34 promoter activity in HBX‐positive cells (Figure 2E,F).

Previous studies show that HBX can interact with CEBP/α to regulate the expression of target genes.^19^, ^20^, ^21^, ^22^ However, whether the interaction of HBX with CEBP/α mediates the activity of IL‐34 promoter is unclear. We measured the interaction of HBX with CEBP/α in HCC cells. The results showed that HBX could bind with CEBP/α in HCC cells using coimmunoprecipitation analysis (Figure 2G). In addition, the colocalization of HBX and CEBP/α was observed in the cytoplasm and nucleus of HBX‐positive cells via immunofluorescence assay (Figure 2H), indicating that HBX interacted with CEBP/α in these locations. Furthermore, we observed that IL‐34 promoter fragment containing CEBP/α binding site could be examined in the anti‐HBX immunoprecipitated candidates using ChIP assay, whereas the interaction of IL‐34 promoter with HBX was significantly decreased when these cells were transfected with CEBP/α shRNA plasmids (Figure 2I). Taken together, these results suggest that HBX could bind to the promoter region of IL‐34 via the interaction with CEBP/α in hepatoma cells.

### 3.3 HBX promotes the expression of CEBP/α via different signal pathways to upregulate IL‐34 in hepatoma cells

**Figure 3:**
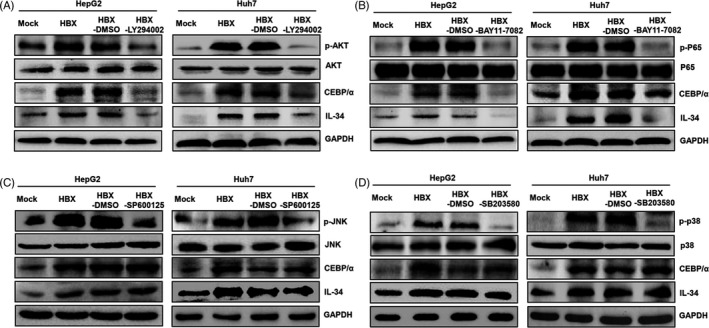
The function of different signal pathways in CEBP/α and IL‐34 expression induced by HBX. A, The function of PI3‐K pathways in the expression of CEBP/α and IL‐34 mediated by HBX. B, The role of the NF‐κB pathways pathway in the expression of CEBP/α and IL‐34 induced by HBX. C, The effect of the JNK pathway in CEBP/α and IL‐34 expression mediated by HBX. D, The function of the p38 pathway in the expression of CEBP/α and IL‐34 induced by HBX. Mock: cells are transfected with the negative control plasmids. HBX: cells are transfected with the HBX plasmids. HBX‐DMSO: the HBX‐positive cells treated with DMSO; HBX‐LY294002: the HBX‐positive cells treated with the inhibitor of PI3‐K pathways; HBX‐BAY11‐7082: the HBX‐positive cells treated with NF‐κB pathway inhibitor; HBX‐SP600125: the HBX‐positive cells treated with the inhibitor of JNK pathways; HBX‐SB203580: the HBX‐positive cells treated with p38 pathway inhibitor

Next, we explored the mechanisms associated with the increase of CEBP/α mediated by HBX to promote IL‐34 expression in HCC cells. Current reports showed that CEBP/α expression is mainly mediated by PI3‐K,^23^ NF‐κB,^24^ JNK and p38 pathways in different cells.^25^ We examined whether HBX was able to promote CEBP/α expression through these pathways. The results showed that HBX could activate PI3‐K, NF‐κB, JNK and p38 pathways in HCC cells (Figure 3). When the cells were treated with the inhibitors of PI3‐K, NF‐κB, JNK and p38 pathways, the activities of AKT (a molecule in PI3‐K pathway), p65 (a molecule in NF‐κB pathway), JNK and p38 were abolished (Figure 3). However, only after the cells treated with PI3‐K and NF‐κB pathway inhibitors, the expression of CEBP/α was significantly suppressed. Meanwhile, IL‐34 expression was also decreased, when these cells were incubated with PI3‐K and NF‐κB pathway inhibitors (Figure 3A,B). Overall, these findings indicated that the activation of PI3‐K and NF‐κB pathways mediated by HBX was responsible for CEBP/α expression to promote the increase of IL‐34 in HCC cells.

### 3.4 IL‐34 contributes to the proliferation and migration of hepatoma cells mediated by HBX

**Figure 4:**
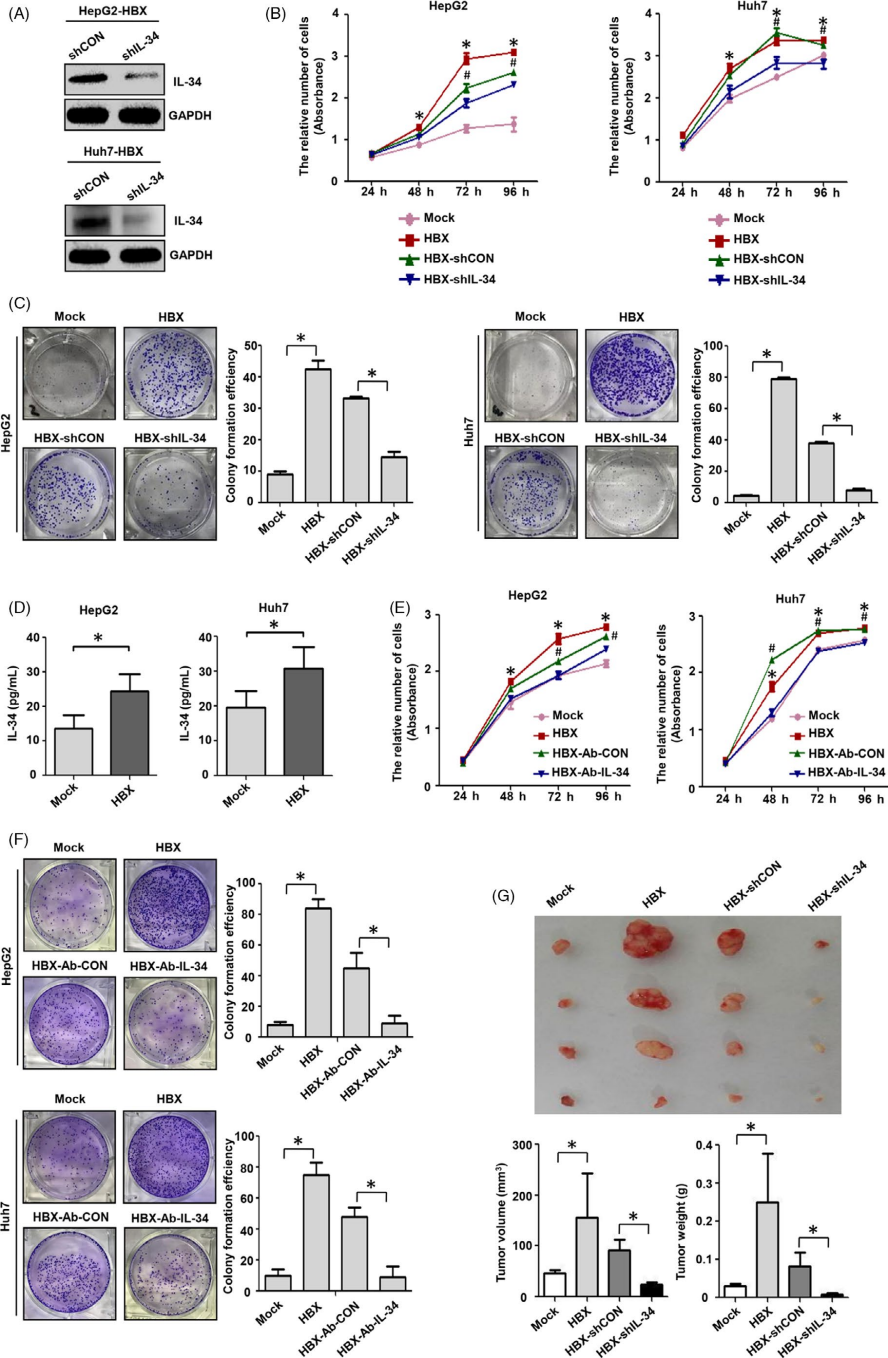
The role of IL‐34 in the proliferation of HCC cells induced by HBX. A, The effect of IL‐34 shRNA in the inhibition of IL‐34 expression in HBX‐positive hepatoma cells. B, The inhibition of IL‐34 expression using shRNA in the proliferation of HBX‐positive HCC cells detected by CCK‐8 assay. C, The inhibition of IL‐34 expression using shRNA in the proliferation of HBX‐positive hepatoma cells examined by plate clone formation assay. D, The expression of IL‐34 in the culture medium of HBX‐positive HCC cells was measured by ELISA. E, The inhibition of IL‐34 function using neutralizing antibody in the proliferation of HBX‐positive HCC cells detected by CCK‐8 assay. F, The inhibition of IL‐34 function with neutralizing antibody in the proliferation of HBX‐positive hepatoma cells examined by plate clone formation assay. G, The role of IL‐34 in the proliferation of HCC cells mediated by HBX in nude mice. shCON: The cells transfected with control plasmid. shIL‐34: The cells transfected with shRNA plasmid against IL‐34. Mock: the cells transfected with negative control plasmid. HBX: the cells transfected with HBX plasmid. HBX‐shCON: The HBX‐positive cells transfected with control plasmid. HBX‐shIL‐34: The HBX‐positive cells transfected with shRNA plasmid against IL‐34. HBX‐Ab‐CON: The HBX‐positive cells treated with control antibody. HBX‐Ab‐IL‐34: The HBX‐positive cells transfected with neutralizing antibody against IL‐34. Compared to Mock, *P < .05; Compared to HBX ‐ shIL‐34, #*P* < .05

Our previous studies indicate that HBX could promote the growth and migration of hepatoma cells.^18^ In addition, current researches suggest that IL‐34 contribute to the proliferation and migration of breast cancer cells.^26^ We examined whether IL‐34 promoted the proliferation and migration of HCC cells mediated by HBX. We firstly constructed a shRNA plasmid targeting IL‐34, and the shRNA was shown to could suppress IL‐34 expression in HBX‐positive cells (Figure 4A). The results of cell viability and plate clonal formation assay suggested that HBX‐positive cells had higher proliferation efficiency than control cells. When HBX‐positive cells were treated with IL‐34 shRNA, the proliferation of hepatoma cells mediated by HBX was declined (Figure 4B,C).

Current studies indicate that, many cytokines, including IL‐17, IL‐6 and IL‐37, could regulate the function of HCC cells by an autocrine manner.^27^, ^28^ We next examined whether HBX could promote the secretion of IL‐34 to mediate the biological function of HCC cells. The results showed that the level of secreted IL‐34 in medium of HBX‐positive cells was higher than that in the medium of control cells (Figure 4D). Next, we added the IL‐34 neutralizing antibody into the medium of HBX‐positive cells and found that when blocking the function of IL‐34 with neutralizing antibody, the proliferation of HBX‐expressing cells was inhibited (Figure 4E,F).

We further determine the effect of IL‐34 on the development of hepatoma cells in vivo. After Huh7‐HBX cells were treated with control or IL‐34 shRNA plasmids for 48 hours, these cells were injected into BALB/c nude mice subcutaneously. We found that the tumour volume and weight of Huh7‐HBX cells were higher than control cells. After transfected with IL‐34 shRNA, the ability of Huh7‐HBX cells form tumours was significantly lower than that of Huh7‐HBX cells transfected with control plasmids in nude mice (Figure 4G). Taken together, these findings suggest that IL‐34 could promote the development of HCC cells induced by HBX in vivo.

**Figure 5:**
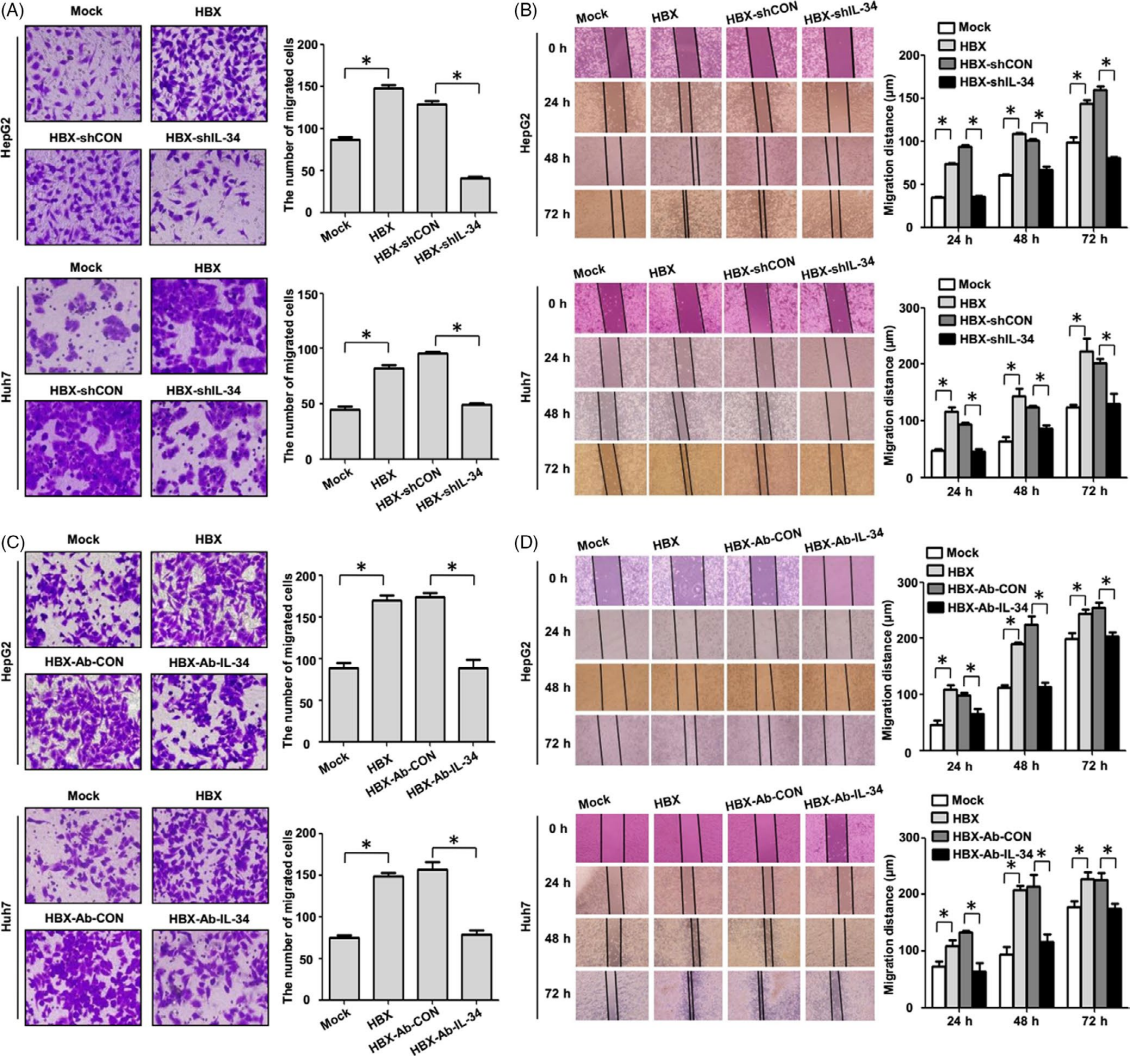
The effect of IL‐34 in the migration of HCC cells mediated by HBX. A, The inhibition of IL‐34 expression using shRNA in the migration of HBX‐positive HCC cells detected by transwell array. B, The inhibition of IL‐34 expression using shRNA in the migration of HBX‐positive hepatoma cells examined by wound healing assay. C, The inhibition of IL‐34 function using neutralizing antibody in the migration of HBX‐positive HCC cells detected with transwell array. D, The inhibition of IL‐34 function using neutralizing antibody in the migration of HBX‐positive hepatoma cells examined by wound healing assay. Mock: the cells transfected with negative control plasmid. HBX: the cells transfected with HBX plasmid. HBX‐shCON: The HBX‐positive cells transfected with shRNA control plasmid. HBX‐shIL‐34: The HBX‐positive cells transfected with shRNA plasmid against IL‐34. HBX‐Ab‐CON: The HBX‐positive cells treated with negative control antibody. HBX‐Ab‐IL‐34: The HBX‐positive cells transfected with neutralizing antibody against IL‐34. *P < .05

Next, we used the transwell and wound healing assay to explore the function of IL‐34 in cell migration mediated by HBX. Consistent with our previous results,^18^ HBX could promote the migration of hepatoma cells. When HBX‐positive cells were treated with IL‐34 shRNA or IL‐34 neutralizing antibody, the migration of hepatoma cells mediated by HBX was inhibited (Figure 5).

### 3.5 IL‐34 promotes the activation of signal pathways and the expression of associated proteins in HBX‐positive HCC cells via CSF1‐R and CD138

**Figure 6:**
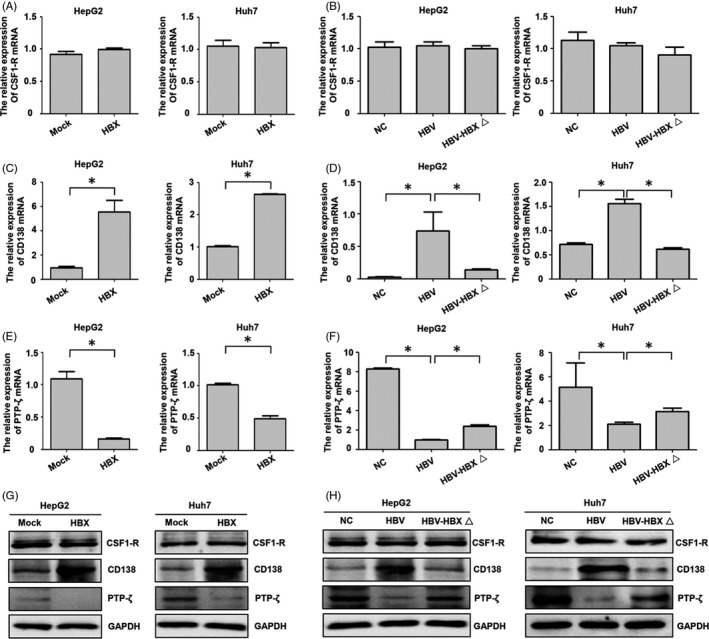
The role of HBX on the expression of CSF1‐R, CD138 and PTP‐ζ in HCC cells. A, The expression of CSF1‐R mRNA in the HBX‐positive HCC cells detected by real‐time PCR. B, The effect of HBV on the expression of CSF1‐R mRNA with or without HBX gene. C, CD138 mRNA expression in the HBX‐positive HCC cells. D, The effect of HBV on the expression of CD138 mRNA with or without HBX gene. E, The expression of PTP‐ζ mRNA in the HBX‐positive HCC cells. F, The effect of HBV on CSF1‐R, CD138 and PTP‐ζ mRNA expression with or without HBX gene. G, The expression of CSF1‐R, CD138 and PTP‐ζ protein in the HBX‐positive HCC cells. H, The effect of HBV on CSF1‐R, CD138 and PTP‐ζ protein expression with or without HBX gene. Mock: the cells transfected with negative control plasmid. HBX: the cells transfected with HBX plasmid. NC: the HCC cells transfected with negative control plasmid; HBV: the HCC cells transfected with HBV plasmid. HBV‐HBXΔ: The cells transfected with HBV plasmid with HBX mutation. *P < .05

Three receptors, including CSF1‐R, CD138 and PTP‐ζ, were found to bind with IL‐34.^12^, ^13^ We detected the mRNA and protein expression of these receptors in HBX and HBV‐positive cells. The results indicated that HBX and HBV had no effect on CSF1‐R. Both HBX and HBV could upregulate CD138 but downregulate PTP‐ζ in HCC cells. In addition, the role of HBV in increasing the expression of CD138 but inhibiting PTP‐ζ expression was mainly dependent on HBX (Figure 6). These results suggest that the effect of IL‐34 on HCC cells might be dependent on the interaction with CD138 or CSF1‐R in HCC cells.

**Figure 7:**
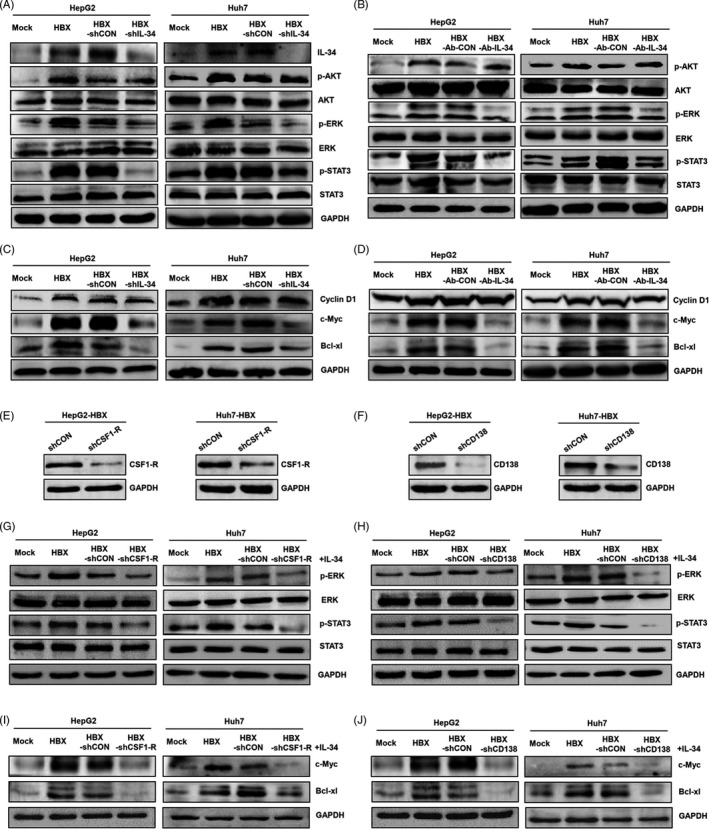
The effect of IL‐34 on intracellular pathways and molecules in HBX‐positive hepatoma cells via CSF1‐R and CD138. A, The inhibition of IL‐34 expression mediated by shRNA in the activation of PI3‐K, ERK and STAT3 signal pathways in hepatoma cells stably transfected with HBX. B, The inhibition of IL‐34 expression mediated by shRNA in the expression of cyclin D1, c‐Myc and Bcl‐xl proteins in hepatoma cells stably transfected with HBX. C, The inhibition of IL‐34 function mediated by neutralizing antibody in the activation of PI3‐K (AKT), ERK and STAT3 signal pathways in HBX‐positive hepatoma cells. D, The inhibition of IL‐34 function mediated by neutralizing antibody in the expression of cyclin D1, c‐Myc and Bcl‐xl proteins in hepatoma cells stably transfected with HBX. E, The effect of CSF1‐R shRNA in the inhibition of CSF1‐R expression in HBX‐positive HCC cells. F, The effect of CD138 shRNA in the inhibition of CD138 expression in HBX‐positive HCC cells. G, The inhibition of CSF1‐R expression mediated by shRNA in the activation of ERK, and STAT3 signal pathways in HBX‐positive cells stimulated by 20 ng/mL IL‐34 recombinant proteins. H, The inhibition of CD138 expression mediated by shRNA in the activation of ERK, and STAT3 signal pathways in HBX‐positive cells stimulated by 20 ng/mL IL‐34 recombinant proteins. I, The inhibition of CSF1‐R expression mediated by shRNA in the expression of c‐Myc and Bcl‐xl proteins in HBX‐positive cells stimulated by 20 ng/mL IL‐34 recombinant proteins. J, The inhibition of CD138 expression mediated by shRNA in the expression of c‐Myc and Bcl‐xl proteins in HBX‐positive cells stimulated by 20 ng/mL IL‐34 recombinant proteins. Mock: the cells transfected with negative control plasmid. HBX: the cells transfected with HBX plasmid. HBX‐shCON: The HBX‐positive cells transfected with shRNA negative control plasmid. HBX‐shIL‐34: The HBX‐positive cells transfected with shRNA plasmid against IL‐34. HBX‐shCSF1‐R: The HBX‐positive cells transfected with shRNA plasmid against CSF1‐R. HBX‐shCD138: The HBX‐positive cells transfected with shRNA plasmid against CD138. HBX‐Ab‐CON: The HBX‐positive cells treated with negative control antibody. HBX‐Ab‐IL‐34: The HBX‐positive cells transfected with neutralizing antibody against IL‐34

Previous study showed that CD138 could enhance the activation of CSF1‐R signal pathways mediated by IL‐34,^29^ implying that IL‐34 may mainly activate the downstream signal pathways and associated molecules of CSF1‐R when interacted with CSF1‐R and CD138 in HCC cells. PI3‐K, ERK and STAT3 pathways are downstream signal pathways of CSF1‐R,^30^, ^31^, ^32^ and we analysed the effect of HBX and IL‐34 on the activation of these pathways. The results showed that the phosphorylation levels of AKT, ERK and STAT3 were increased in HBX‐positive cells. After treated HBX‐positive cells with IL‐34 shRNA or IL‐34 neutralizing antibody, the activation levels of ERK and STAT3, but not AKT were significantly inhibited (Figure 7A,B). Together, these results suggested that IL‐34 contributed to the activation of ERK and STAT3 signal pathways mediated by HBX in hepatoma cells.

We next examined the downstream molecules of CSF1‐R, including CyclinD1, c‐Myc and Bcl‐xl,^32^ in HBX‐positive cells. Our data indicated that the expressions of c‐myc and Bcl‐xl were increased in HBX‐positive cells. After treated HBX‐positive cells with IL‐34 shRNA or neutralizing antibody, the expressions of c‐Myc and Bcl‐xl were significantly decreased (Figure 7C,D). Taken together, these results suggested that the elevated IL‐34 mediated by HBX was associated with the expression of c‐Myc and Bcl‐xl in HCC cells.

Furthermore, we constructed the shRNA plasmids against CSF1‐1R and CD138 to explore whether the activation of signal pathways and the expression of associated molecules mediated by IL‐34 were dependent on CSF1‐1R and CD138. As shown in Figure 7E,F, the shRNAs could significantly inhibit the expressions of CSF1‐1R and CD138 in HBX‐positive HCC cells. In addition, consistent with our expectation, after treated HBX‐positive HCC cells with CSF1‐1R and CD138 shRNAs, the activation of EKR and STAT3 and expression of c‐Myc and Bcl‐xl in HBX‐positive HCC cells stimulated by IL‐34 were significantly inhibited (Figure 7G‐J).

### 3.6 IL‐34 promotes the proliferation and migration of HBX‐positive hepatoma cells via CSF1‐R and CD138

**Figure 8:**
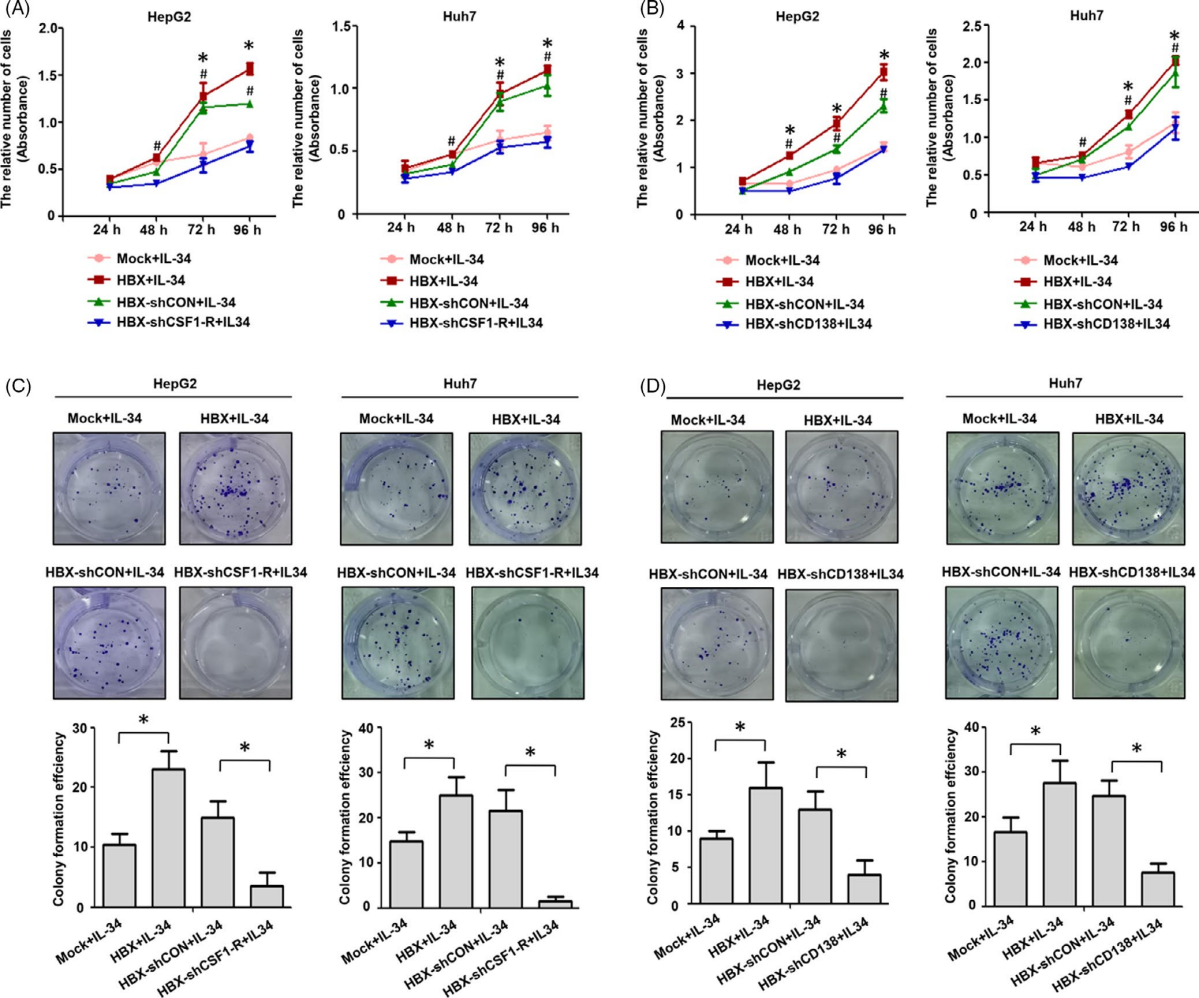
IL‐34 promotes the proliferation of HBX‐positive HCC cells via CSF1‐R and CD138. A, The inhibition of CSF1‐R expression using shRNA in the proliferation of HBX‐positive HCC cells stimulated by 20 ng/mL IL‐34 and detected by CCK‐8 assay. B, The inhibition of CD138 expression using shRNA in the proliferation of HBX‐positive HCC cells stimulated by 20 ng/mL IL‐34 and detected by CCK‐8 assay. C, The inhibition of CSF1‐R expression using shRNA in the proliferation of HBX‐positive hepatoma cells stimulated by 20 ng/mL IL‐34 and examined by plate clone formation assay. D, The inhibition of CD138 expression using shRNA in the proliferation of HBX‐positive hepatoma cells stimulated by 20 ng/mL IL‐34 and examined by plate clone formation assay. Compared to Mock + IL‐34, *P < .05; Compared to HBX‐shCSF1‐R + IL‐34, #*P* < .05; Compared to HBX‐shCD138 + IL‐34, #*P* < .05

**Figure 9:**
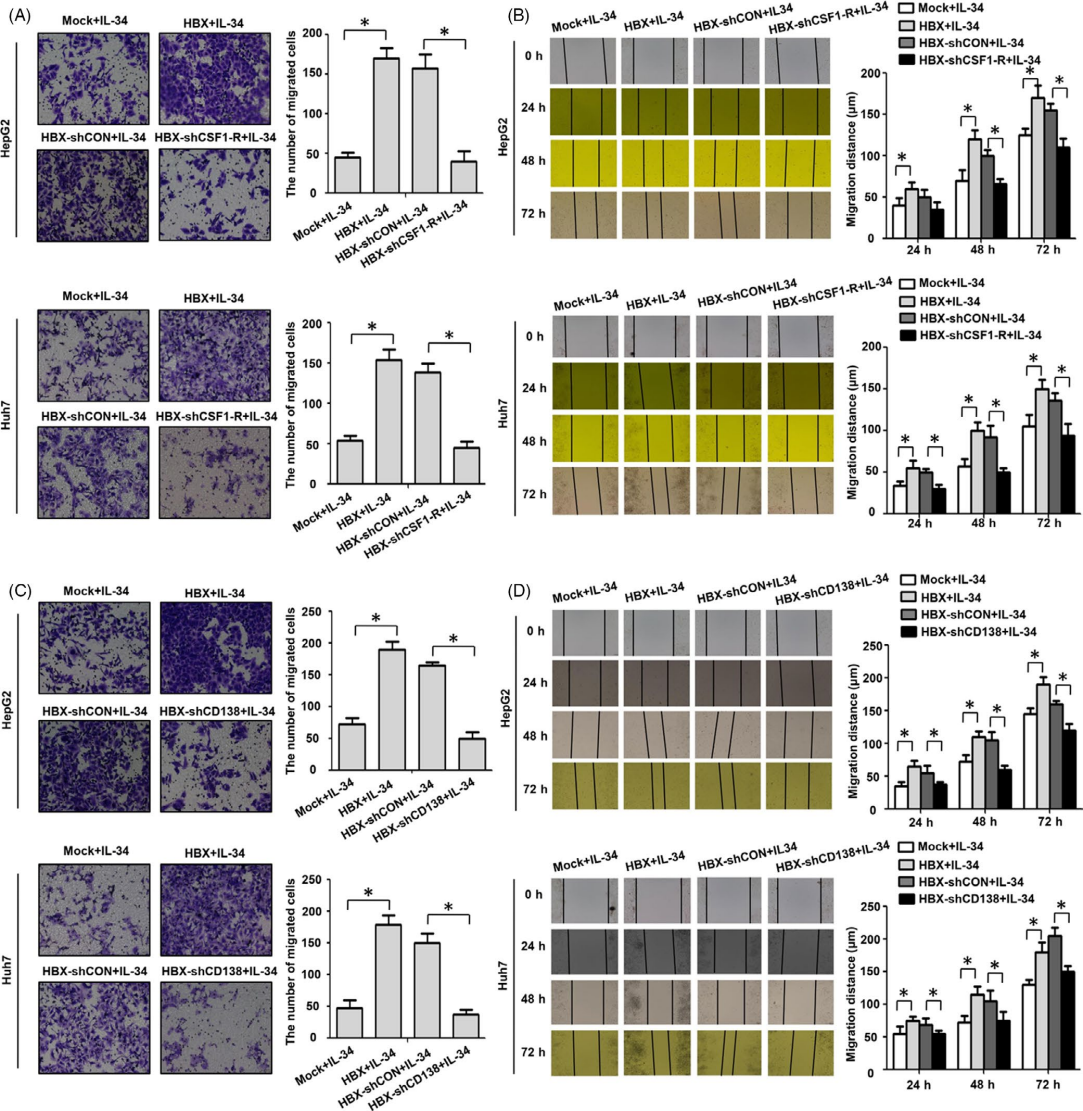
IL‐34 promotes the migration of HBX‐positive HCC cells via CSF1‐R and CD138. A, The inhibition of CSF1‐R expression using shRNA in the migration of HBX‐positive HCC cells stimulated by 20 ng/mL IL‐34 and detected by transwell array. B, The inhibition of CSF1‐R expression using shRNA in the migration of HBX‐positive hepatoma cells stimulated by 20 ng/mL IL‐34 and examined by wound healing assay. C, The inhibition of CD138 expression using shRNA in the migration of HBX‐positive HCC cells stimulated by 20 ng/mL IL‐34 and detected by transwell array. D, The inhibition of CD138 expression using shRNA in the migration of HBX‐positive hepatoma cells stimulated by 20 ng/mL IL‐34 and examined by wound healing assay. *P < .05

We next assessed whether IL‐34 promotes the proliferation and migration of HBX‐positive HCC cells via CSF1‐R and CD138. The results of cell viability and plate clonal formation assay suggested that HBX‐positive cells had higher proliferation efficiency than control cells stimulated by IL‐34 (Figure 8). After HBX‐positive cells treated with CSF1‐R and CD138 shRNAs, the proliferation of HBX‐positive HCC cells stimulated by IL‐34 was inhibited. In addition, the results of the transwell and wound healing assay showed that, compared to control cells, a higher migration efficiency of HBX‐positive cells stimulated by IL‐34 was observed. When HBX‐positive cells were treated with CSF1‐R and CD138 shRNAs, the migration of HBX‐positive cells stimulated by IL‐34 was declined (Figure 9).

## 4 DISCUSSION

IL‐34 is considered to be associated with HBV infection and HCC.^14^, ^15^, ^16^ However, the role and associated mechanisms of IL‐34 in HBV‐related HCC are not well clarified. In this study, we found that HBV contribute to the increase of IL‐34 via HBX in HCC cells. Furthermore, the role of HBX on IL‐34 expression is mainly dependent on CEBP/α, and HBX could activate different signal pathways for the upregulation of CEBP/α to facilitate IL‐34 expression. Furthermore, via CSF1‐R and CD138, IL‐34 enhanced the proliferation and migration of HCC cells, and promoted the activation of signal pathways and the expression of associated proteins mediated by HBX.

As a newly identified cytokine, IL‐34 is produced by a wide range of cells and participates in the survival, proliferation and differentiation of various cells.^12^, ^13^ Currently, the expression of IL‐34 is identified as increase in patients with infection of various viruses, including HIV,^33^ influenza A virus^34^ and HCV.^35^ Though the results from Cheng et al suggested that the expression of serum IL‐34 was significantly decreased in CHB patients and this cytokine could inhibit the replication of HBV,^14^ the increase of IL‐34 was reported by Wang et al in CHB patients, especially in HBeAg negative patients with high HBV DNA levels.^15^ Importantly, the increased IL‐34 was associated with liver inflammation and fibrosis in HBV infection, and the results were consistent with the phenomenon observed in non‐alcoholic fatty liver disease and chronic hepatitis C.^35^, ^36^ Besides these, IL‐34 is shown to increase in different tumour cells,^26^, ^37^, ^38^, ^39^ and related to the prognosis of various cancers. In addition, Zhou S et al showed that the upregulation of IL‐34 contributed the development of HCC.^16^ Around the world, HBV is a major cause for the development of HCC. Via RNA‐Seq based transcriptome analysis, HBV was found to could increase the expression of IL‐34 in Huh7 cells, which was shown in the supplementary data of published article from Jagya N et al^40^ In the study, we are interested in whether IL‐34 participating in the hepatocarcinogenesis mediated by HBV. We measured the expression of IL‐34 mediated by the virus, and the results showed that HBV could increase IL‐34 expression in hepatoma cells. In addition, compared to CHB and HBV‐negative HCC patients, the levels of serum IL‐34 in HBV‐related HCC patients were increased. Furthermore, IL‐34 expression in HBV‐related tumour tissues was higher than those in HBV‐positive adjacent tissues, HBV‐negative HCC and HBV‐negative adjacent tissues. Among the encoded genes in HBV genome, only HBX was found to induce the expression of IL‐34 in HCC cells. Up to date, HBX has been demonstrated to be a very important cancer cofactor and could mediate the development of HCC through modulating multiple host factors to influence the biological functions of HCC cells.^5^, ^6^ In this study, our results suggest that IL‐34 was associated with the development of HCC mediated by HBX.

Next, we detect the mechanisms of IL‐34 mediated by HBX, the results showed that HBX could promote IL‐34 expression at the promoter level, and the core region of IL‐34 promoter mediated by HBX was screened out. Based on bioinformatics analysis and the sequence mutation in predicted binding site, CEBP/α was found to facilitate the activation of IL‐34 promoter mediated by HBX. Furthermore, we observed that inhibition of CEBP/α expression by shRNA, the expression of IL‐34 induced by HBX was significantly decreased. Current studies indicated that CEBP/α participated in regulating the expression of multiple genes,^19^, ^20^ including FABP1 and SOCS3 that mediated by HBX in HCC. Moreover, previous studies show that HBX could interact with CEBP/α,^21^, ^22^ and the interaction help HBX bind to special regions of the promoters to mediate the expression of target genes. Consistent with these reports, our results indicated that HBX could bind to CEBP/α, and the collocation of HBX and CEBP/α was found in the cytoplasm and nucleus of HCC cells. Furthermore, HBX was also found to bind to the IL‐34 promoter through the interaction with CEBP/α. Together, our results confirmed that CEBP/α played an important role in IL‐34 expression mediated by HBX, and HBX mainly induced the expression of IL‐34 via upregulation and interaction with CEBP/α.

Previous researches show that multiple signal pathways, including PI3‐K, NF‐κB, JNK and p38, contribute to the expression of CEBP/α in different cells.^23^, ^24^, ^25^ Furthermore, HBX could induce the activation of these pathways to regulate the expression of various cellular factors in HCC cells.^17^, ^41^, ^42^ In addition, JNK and NF‐κB pathways have been reported to induce the expression of IL‐34 in cells with multiple types.^43^, ^44^ However, whether these signal pathways are associated with the expressions of CEBP/α and IL‐34 mediated by HBX is unknown. Consistent with previous reports,^17^, ^41^, ^42^ we found that HBX could induce the activation of these pathways as mentioned above. Furthermore, our results showed that PI3‐K and NF‐κB pathways were responsible for IL‐34 expression mediated by HBX, and the increase of IL‐34 induced by PI3‐K and NF‐κB pathways was dependent on CEBP/α in hepatoma cells.

Though Zhou S et al show that IL‐34 could mediate the proliferation and migration of HCC cells via macrophages,^16^ current researches indicated that IL‐34 could directly regulate the proliferation and migration of tumour cells.^26^ We explored whether HBX could mediate the function of HCC cells through IL‐34. Consistent with our previous studies,^18^, ^45^ HBX could promote the growth and migration of HCC cells. Furthermore, when knocking down of IL‐34 by shRNA, the proliferation and migration efficiency of HCC cells mediated by HBX was decreased. Previous studies show that multiple autocrine cytokines, including IL‐17 and IL‐37 , could regulate the biological function of hepatoma cells.^27^, ^28^ We next investigated whether HBX could promote the secretion of IL‐34 from HCC cells and the secreted IL‐34 in turn had an effect on HCC cells. As expected, compared with control group, the increased expression of IL‐34 was found in the medium of HBX‐positive cells. When we added the neutralizing antibody targeting IL‐34 into the medium of HBX‐positive cells, the proliferation and migration abilities of these cells were decreased. These results indicated that autocrine IL‐34 could directly mediate the biological function of HCC cells induced by HBX. In addition, we further explore the function of IL‐34 on HCC mediated by HBX in vivo environment. We found that HBX could promote the growth of HCC cells in nude mice, and these results were coincided with reported studies.^46^, ^47^ Furthermore, after inhibited IL‐34 expression in HBX‐expressing HCC cells, the growth ability of HCC was decreased in vivo. These results indicated that IL‐34 is a potential therapeutic target for HBV‐related HCC in vivo environment.

Current studies showed that, binding to its receptors, IL‐34 could mediate the activation of intercellular signal pathways such as PI3‐K, ERK and STAT3, and induce the expression of the downstream molecules to regulate the function of target cells.^48^, ^49^ In the study, we detected the expression of IL‐34 receptors, including CSF1‐R, CD138 and PTP‐ζ, in HBX and HBV‐positive cells. The results showed that HBX or HBV had no significantly role on CSF1‐R expression. Dependent on HBX, HBV could upregulate CD138 but downregulate PTP‐ζ in HCC cells. These results suggested that IL‐34 may stimulate HCC cells via binding to CD138 or CSF1‐R. Previous study indicated that, CD138 contribute to the activation of downstream signal pathway of CSF1‐R, when binding with IL‐34.^29^ Besides, PI3‐K, ERK and STAT3 signal pathways are reported downstream pathways of CSF1‐R.^30^, ^31^ In addition, cyclinD1, c‐Myc and Bcl‐xl are the downstream molecules of CSF1‐R.^32^ In the study, we detected whether IL‐34 could activate these signal pathways and associated downstream molecules in HBX‐positive cells. Our results suggested that HBX could induce the activity of ERK and STAT3 pathways and promote the expression of c‐Myc and Bcl‐xl through IL‐34. AKT is a downstream factor of CSF1‐R.^30^, ^31^ Though we found that HBX could activate AKT, the inhibition of CSF1‐R function mediated by IL‐34 could not suppress the AKT activation in HBX‐positive HCC cells. Current studies have been reported that multiple factors, including Notch1 and miR‐29a, mediated by HBX could activate AKT,^50^, ^51^ and maybe these factors but not CSF1‐R mediated by IL‐34 played a dominant role on the activation of AKT in HBX‐positive HCC cells. Previous reports have shown that ERK and STAT3 pathways are important signal pathways that involved in the development of HCC.^52^, ^53^ In addition, c‐Myc is an oncogene that participates in the growth of HCC cells.^54^ Bcl‐xl is found to mediate the survival of HCC cells.^55^ These results indicate that IL‐34 mediated by HBX may regulate the progress of HCC via multiple signal pathways and associated molecules during HBV infection.

We further assessed whether the activation of signal pathways and the increase of intercellular molecules mediated by IL‐34 in HBX‐positive cells were dependent on CSF1‐R and CD138. Expectedly, our results suggested that both the activation of ERK and STAT3 and the expression of c‐Myc and Bcl‐xl induced by IL‐34 were mainly relied on CSF1‐R and CD138 in HBX‐expressing HCC cells. We also explored whether IL‐34 could regulate the biological function of HBX‐positive HCC cells via CSF1‐R and CD138. Compared to control cells, the growth and migration efficiency of HBX‐positive HCC cells stimulated by IL‐34 were enhanced. After knocking down of CSF1‐R and CD138 by shRNA, the efficiency of proliferation and migration of HBX‐positive HCC cells stimulated by IL‐34 was declined.

In conclusion, our study reveals that HBX could promote the expression of IL‐34 via CEBP/α in HCC cells. Via CSF1‐R and CD138, IL‐34 plays important roles in the growth and migration of HCC cells mediated by HBX, and the effect of IL‐34 on HCC cells is associated with intracellular signal pathways and associated molecules. Our study provides a further understanding on the role of IL‐34 in HBX‐mediated hepatocarcinogenesis and brings a potential therapeutic target for HBV‐related HCC. In addition, although our results indicate that HBV could induce IL‐34 expression to promote the growth and migration HCC cells directly, current studies show that IL‐34 are also involved in the differentiation and activation of multiple immune cells, including macrophages, monocytes and dendritic cells,^16^, ^39^, ^56^ and these immune cells maybe also participate in the progression of HCC mediated by IL‐34 in vivo. Future works are needed to explore the role of different immune cells that could be activated by IL‐34 in the development of HCC, to comprehensively understand the importance of IL‐34 in the development of HBV‐related HCC.

## CONFLICT OF INTEREST

The authors declare no competing interests.

## Data Availability

The data that support the findings of this study are available from the corresponding author upon reasonable request.
