# Supplementary material for: Interleukin‐34 mediated by hepatitis B virus X protein via CCAAT/enhancer‐binding protein α contributes to the proliferation and migration of hepatoma cells
Source: Cell Prolif. 2019 Oct 16;52(6):e12703. doi: 10.1111/cpr.12703 (PMC6869657; doi:10.1111/cpr.12703)
Supplement: Supplementary file 1 [file CPR-52-e12703-s001.docx]

**SUPPLEMENTARY METHODS**

**Reagents, plasmids and clinical samples**

Antibodies against HBX, phosphorylated JNK (p-JNK), JNK, p-AKT, AKT, GAPDH, β-actin, goat anti-mouse IgG-HRP, rabbit anti-goat IgG-HRP, and goat anti-rabbit IgG-HRP antibodies were collected as mentioned before^17^. IL-34, C/EBPα antibodies were purchased from Abcam (Cambridge, MA, USA). Antibody against HBsAg (HBS) was purchased from Santa Cruz Biotechnology (Santa Cruz, CA, USA). Antibodies against p-p38, p38, p-ERK, ERK, p-STAT3, STAT3, PNCA, Cyclin D1, c-Myc, Bcl-xl were from Proteintech (Wuhan, Hubei, China). NF-κB inhibitor BAY11-7082, JNK pathway inhibitor SP600125, PI3-K pathway inhibitor LY294002 and p38 pathway inhibitor SB203580 were from Sigma Aldrich (St. Louis, MO, USA). IL-34 neutralizing antibody was purchased from R&D Systems (Minneapolis, MN, USA). G418, Trizol reagent, Clarity western ECL substrate, Lipofectamin 2000, TIANScript RT Kit and EpiQuik chromatin immunoprecipitation kit were obtained as preciously described^17^. Human IL-34 recombinant protein was purchased from PeproTech (Rocky Hill, NJ, USA). LipoMax DNA transfection reagent was obtained from Sudgen Biotechnology (Nanjing, Jiangsu, China). Matrigel solution was from BD Biosciences (San Diego, CA, USA).

HBX, HBS, HBC, HBe, HBP, preS2, and preS1 plasmid, pUC18-HBV1.2 (HBV plasmid), and pUC18-HBV1.2-ΔHBX (HBV plasmid with the full HBX gene deletion mutant), and short hairpin RNA (shRNA) plasmids against HBX, pGPU6/GFP/Neo-HBX, were obtained as described previously^44^. The plasmid containing shRNA against IL-34, pGPU6/GFP/Neo-IL34, shRNA vector against CEBP/α, pGPU6/GFP/Neo-CEBP/α, shRNA vector against CSF1-R, pGPU6/GFP/Neo-CSF1-R, shRNA vector against CD138, pGPU6/GFP/Neo-CD138, and control vectors pGPU6/GFP/Neo were purchased from GenePharma Co, Ltd (Suzhou, Jiangsu, China).

The serums of health controls (NC, n=20), CHB patients (n=30), HCC patients with HBV infection (HBV-HCC, n=40), and HBV-negative HCC patients (non-HBV-HCC, n=30) were collected from the department of infectious diseases and department of laboratory medicine, affiliated hospital of Xuzhou medical university. HBV-negative adjacent tissues (n=30), HBV-negative HCC (n=30), HBV positive adjacent tissues (n=30) and HBV-related HCC tissues (n=50) were collected from the department of pathology, affiliated hospital of Xuzhou medical university and purchased from Shanghai Outdo Biotech Co., Ltd. The present study was approved by the ethics committee of Xuzhou medical university, and the informed consent was obtained from all patients.

**Cell transfection**

Using Lipofectamin 2000 or LipoMax DNA transfection reagents followed by the instruction, the cells were transfected with different plasmids. In addition, the cells stably transfected with HBX plasmids and control plasmids were selected with G418 as mentioned previously^17,18^.

**RT-PCR and real-time PCR**

RT-PCR and real-time PCR were followed as described before^20^. Briefly, total cellular RNA was collected via Trizol reagent. Reverse transcription was performed with TIANScript RT Kit. The primer sequences for GAPDH as well as the amplification conditions used for PCR were described previously^20^. Primers for IL-34, CSF-1R, CD138, and PTP-ζ were ACATGAAACACTACTTCCCCATCA and GGCTCACC AAGACCCACAGA, CTCAGCACCAACAACGCTACC and CAGACAGGGCAGT AGTGCGTC, CTTCACCTTTGAAACCTCGGG and AGGCACACAGCAAAGAT GAGC, CACATATGAACTTGTCGGGGAC and ATATATACCCTTGGGATATGTT CTCAG. The conditions for amplification were: 2 min at 94 °C followed by 30 s at 94 °C, 30 s at 60 °C or 62 °C, and 30s at 72 °C for 45 cycles. The relative expression levels of different genes were normalized to GAPDH gene.

**Construction of the plasmids with different regions of IL-34 promoter**

Six different regions of the IL-34 promoter were cloned into the PGL3 plasmid. Briefly, using specific primers, six different 5′-flanking regions (-2000/+155, -924/+155, -839/+155, -745/+155, -491/+155 and -134/+155) of IL-34 were amplified and inserted into the KpnI/BglII site of pGL3-Basic vector, and were named as PGL3-P (-2000/+155), PGL3-P (-924/+155), PGL3-P (-839/+155), PGL3-P (-745/+155), PGL3-P (-491/+155) and PGL3-P (-134/+155). The forward primers for the IL-34 promoter with different regions were as follows: GGGGTACCTGG GTGTTAATAAGGGGTTCCTAAATC, GGGGTACCGGTTGTGATTGTGCCACT GCTCTCT, GGGGTACCTGTAATCCCAGCACTTGGGAGGCT, GGGGTACCAC CTTTTGGAGCTCATGTCTCTTCATC, GGGGTACCCATCACTCAGTTTTGTGT TTTGTTTCG, GGGGTACCTGGCGGGTCCTCTGGGTTGAA and the reverse primer was GAAGATCTCTGTTCACGTGCCAAGACTCGGTC. The PGL3-P (-745/+155) mutant, which carried a substitution of seven nucleotides (GTAACCA) in the potential binding sites of C/EBPα (CATTGGT) in IL-34 promoter region, was constructed with the overlapping extension PCR as described^17^.

**Luciferase reporter gene assays**

The manipulation of the luciferase reporter gene assays was described in detail before^17^. Briefly, 1 × 10^5^ HBX expressing cells or control cells were placed into plates with 24 well. After 24 h, the plasmids of different regions of IL-34 promoter or the pGL3-Basic plasmid were cotransfected with the pRL-TK plasmid into cells. After 24 h, the cells were collected and lysed in 1× passive lysis buffer. Followed by the instruction, the results were measured via the dual luciferase reporter assay system as described^17^.

**ELISA**

The concentrations of IL-34 in serum or culture medium in different groups were detected by human IL-34 ELISA kit (Cusabio Biotech, Wuhan, Hubei, China). The detection of IL-34 was in accordance with the manufacturer’s operating manual.

**Co-IP**

The manipulation of Co-IP was as followed as described^17^. At first, HBX positive cells and control cells were collected and lysed. Next, the protein extracts were incubated with HBX or CEBP/α antibodies, and Protein G Sepharose beads for 12 h at 4 °C. Immunoglobulin G (IgG) was used in the negative control group. After immunoprecipitates were washed with the lysis buffer for five times, the proteins were separated using sodium dodecyl sulfate-polyacrylamide gel electrophoresis, and were further immunoblotted with specific antibodies against HBX or CEBP/α. Last, the results were visualized with Clarity™ ECL Western Blot substrate.

**Western blot analysis**

The protocols for western blot have been described^19^. Briefly, total proteins were extracted using cell lysis, subjected to sodium dodecyl sulfate polyacrylamide gel electrophoresis (SDS-PAGE) and transferred onto polyvinylidene difluoride (PVDF) membranes. After blocked using 5 % milk in Tris-buffered saline for 2 h at room temperature, the PVDF membranes were incubated with different primary antibodies at 4 °C overnight. Then, the membranes were incubated with HRP-conjugated secondary antibodies at room temperature for 2 h. Protein bands were detected with Clarity™ ECL Western Blot substrate.

**Immunohistochemistry (IHC) analysis**

The IHC was performed in details as previously described^18^. Briefly, after fixed with 4% formaldehyde, all target tissues were embedded in paraffin and sectioned. Next, tissue sections were deparaffinized and rehydrated. In order to retrieve the antigen, the tissue sections were incubated in 0.01 M sodium citrate. Sequentially, the tissue sections were treated with 3% H2O2 and blocked with 10% goat serum for 1 h. The tissue sections were next incubated with IL-34 antibodies overnight. Then, HRP-conjugated antibodies were further added to tissue sections for 2 h. When the staining of tissue sections was appeared after incubated with 3,3′-diaminobenzidine (DAB), double distilled water was utilized to terminate the reaction. Finally, the sections were counterstained using hematoxylin, and the results were detected via Olympus microscopy (200×). Additionally, the expression of IL-34 in target tissues was dependent on two parameters: the proportion of stained tumor cells as well as staining intensity, which was described previously^17^.

**Immunofluorescence analysis**

The location of HBX and CEBP/α in HCC cells was measured by immunofluorescence as described^19^. Briefly, the target cells were seeded on coverslips for 24 h in 24-well plates. After the coverslips were fixed with ice-cold acetone for 10 min, and blocked with 5% bovine serum albumin (BSA) in phosphate-buffered saline (PBS) for half an hour, the coverslips were incubated with HBX and CEBP/α antibodies for 12 h at 4 °C. Next, the coverslips were washed with PBS for 3 times and then incubated with Alexa Fluor 488- and Alexa Fluor 594-conjugated antibodies for 2 h at room temperature. Cell nuclei were stained with DAPI for 10 min. The results were acquired using the Olympus fluorescence microscope (400×).

**ChIP**

The ChIP assay was performed via EpiQuik chromatin immunoprecipitation kit as described[^17^](#_ENREF_17). Briefly, HBX positive cells were transfected with shRNA-CEBP/α plasmid or control shRNA plasmid for 48 h and then fixed with 1% formaldehyde. The complexes of proteins with DNA were immunoprecipitated with HBX antibody or CEBP/α antibody. Normal mouse IgG was used as negative control antibody. The IL-34 gene promoter from target samples was measured by PCR with IL-34 promoter-specific primers (TCTCTTCATCTGTAGACTGGGTAACT and AAACTG AGTGATGTGGTGGTGAC). An input control was utilized, in which the soluble chromatin prior to immunoprecipitation was amplified.

**Cell viability assay**
The cell viability assay was followed as described^19^. Briefly, 100 μL cell suspension was placed into 96-well plates with six parallel wells in each group. After the cells were incubated for 24, 48, 72, and 96 h, the number of cells was measured via CCK-8 kit. Using the ClinBio-128 plate reader (SLT, Austria), the relative proliferation rate of cells in different groups was detected by optical density (OD) values at 450 nm.

**Plate clone formation assay**
The HCC cells were added into 6-well plates or 12-well plates with three parallel wells in each group. After incubation for 2 weeks at 37 °C, phosphate buffered saline (PBS) were used to wash all cells twice and crystal violet staining solution was utilized to stain. The efficiency of clone formation was calculated as described^19^.

**Animal transplantation**

The animal experiments were approved by the ethics committee of Xuzhou medical university. Four-week-old female BALB/c nude mice, which were purchased from Shanghai Laboratory Animal Co., Ltd (Shanghai, China), were housed in specific pathogen-free and temperature-controlled conditions (22±2℃) with a 12 h ligh/dark cycle. After transfection with IL-34 shRNA or control plasmids for 48h in Huh7-HBX cells, the cells were harvested and resuspended at a concentration of 1×10^7^/ml in sterile PBS. Then the null mice were injected with 0.1 ml of the cell suspension plus 0.1 ml Matrigel solution at the shoulder in each group (n=4). Tumor was evaluated by measuring the length and width using calipers, and the volume of tumors was calculated with the following formula: (length×width^2^) ×0.5. After 40 days, the mice in all groups were killed and the tumors were excised and measured.

**Transwell array**
The transwell array was manipulated as previously described^19^. Briefly, The cells were resuspended in serum-free medium and placed in the upper chamber of a transwell plate. 1 mL medium with 10 % fetal bovine serum (FBS) was used as chemoattractant and placed at bottom chamber. The cells in different groups were incubated at 37 °C for 24 h. The cells which did not pass through the polycarbonate membrane were scraped off using a cotton swab to remove. The cells that passed over the polycarbonate membrane were considered as migrated cells. The migrated cells were fixed with 4 % paraformaldehyde, stained using crystal violet and counted.

**Wound healing assay**The cells in each group were plated in 6-well plates or 12-well plates and cultured to reach 90 % confluence. Then, a wound was created using a micropipette tip on each plate. Images were collected via a microscope (×400), and migration distances in different groups were calculated as followed described previously^19^.

**Statistical analysis**

The data were presented as means ±standard deviation (SD), and were analyzed by t test, one-way ANOVA, or Mann-Whitney test where appropriate. The analysis on the relative expression of IL-34 detected by IHC was used with chi-square test. A p < 0.05 was considered statistically significant.
